# Identification and validation of T cell senescence-related prognostic genes in gastric carcinoma and investigation of their potential regulatory mechanisms

**DOI:** 10.1007/s12672-025-02477-4

**Published:** 2025-06-02

**Authors:** Jing Liu, Huiyu Wang, Fang Huang, Zhiqiang Hang, Pan Xu, Runjie Wang, Junying Xu

**Affiliations:** Department of Oncology, The Affiliated Wuxi People’s Hospital of Nanjing Medical University, Wuxi People’s Hospital, Wuxi Medical Center, Nanjing Medical University, 299 Qingyang Road, Liangxi District, Wuxi, 214000 Jiangsu Province China

**Keywords:** Gastric carcinoma, Immunosenescence, Prognostic genes, Risk model, Single cell RNA sequencing data analysis

## Abstract

**Objective:**

This study aimed to identify prognostic genes associated with immunosenescence in gastric carcinoma (GC) and to elucidate their mechanisms to provide new ideas for the clinical treatment of GC.

**Methods:**

According to single cell data, clustering and annotation were conducted to acquire key cells. Then, differentially expressed genes (DEGs) in key cells (KC-DEGs) and TCGA-GC (GC-DEGs) were obtained, and took their intersection with CS-RGs to obtain candidate genes. Afterwards, prognostic genes were identified by regression analyses. Following this, the risk model was constructed, and the high-risk and low-risk groups were obtained. Next, a nomogram based on independent prognostic factors was constructed for predicting survival in GC. Finally, to further explore the mechanisms associated with the risk groups, immune microenvironment analysis was performed.

**Results:**

T cells were used as key cells. Subsequently, AXL, PIM1, STK40, CXCL1, IFNG and SERPINE1 were identified as prognostic genes. The risk model and nomogram had favourable predictive capability in survival of GC patients. Surprisingly, 17 differential immune cells had higher levels of infiltration in the high-risk group, a result that was further confirmed in tumor purity. Notably, there was mostly a positive correlation between them and prognostic genes. Then, both tumor mutation burden (TMB) and microsatellite instability (MSI) were lower in the high-risk group, suggested the high-risk group might be associated with lower treatment benefit.

**Conclusion:**

6 prognostic genes were identified, providing novel concepts in prognosis and therapy for GC.

**Supplementary Information:**

The online version contains supplementary material available at 10.1007/s12672-025-02477-4.

## Introduction

Gastric carcinoma (GC) is one of the most prevalent malignant tumors of the digestive system. It ranks fifth in incidence, accounting for 5.6% of all cancer cases, and fourth in mortality, representing 7.7% of all cancer deaths globally, thereby posing a significant threat to human health [1]. Due to its high aggressiveness and heterogeneity, GC presents a significant challenge in diagnosis and treatment, the median overall survival (OS) for advanced gastric cancer is approximately 8 months [2]. Currently, the primary treatment modalities for GC include surgical resection along with combined therapy like chemotherapy, targeted therapy and immunotherapy, which can significantly improve the prognosis of patients [3, 4]. Particularly, recent research reveals that immunotherapy including PD-1/PD-L1/CTLA-4 immune checkpoint inhibitors has shown good therapeutic effects on GC [5]. However, although these treatment strategies have improved clinical efficacy, the prognosis of GC patients hasn’t been substantially improved due to factors such as toxicity associated with chemotherapeutic agents and difficulties in identifying suitable candidates for targeted therapies and immunotherapies alongside issues related to drug resistance [2]. Therefore, it's urgent to identify more reliable prognostic genes to predict the outcomes for GC patients and explore additional potential therapeutic targets.

The incidence and prevalence of most cancers increase with age due to tumor escape mechanisms and weakened immune surveillance. Immunesenescence, defined as the aging of the immune system, plays a vital role [6]. Reduced T cell production (more highly differentiated T cells instead of naive T cells), changed secretory phenotypes, enhanced glycolysis, and reactive oxygen species production are features of immunesenescence contributing to cancer susceptibility [7]. In the tumor microenvironment (TME), senescent immune cells may help tumors grow and spread in multiple ways, thus affecting the efficacy of immunotherapy, so immunesenescence has drawn more attention [8]. Among various immune cells, T cells recognized as the most effective in eliminating tumor cells have garnered significant attention due to their dysfunction [9]. Increasing evidence indicates that malignant tumors exploit T cell senescence (TCS) as a key strategy for evading immune surveillance [10, 11]. The accumulation of senescent T cells results in ineffective clearance of tumor cells and contributes to immunosuppression within TME, thereby adversely impacting the efficacy of immunotherapy [10]. Consequently, effectively inhibiting and reversing TCS has become a critical objective for enhancing tumor immunotherapy [12]. To date, no studies have investigated the correlation between TCS and GC. Therefore, it is essential to identify target genes and mechanisms associated with TCS in GC.

Based on public databases, this study identified prognostic genes associated with TCS in GC through single-cell analysis, differential expression analysis, machine learning, and univariate Cox regression and LASSO regression analysis. Risk model and nomogram model were constructed to predict the survival of GC patients, and the molecular regulatory mechanism of high and low risk groups was explored. This study provides a new idea for the prognosis and treatment of GC.

## Materials and methods

### Data source

The RNA sequencing data for 407 gastric tissue samples, containing 375 GC samples and 32 normal samples from individuals, came from The Cancer Genome Atlas (TCGA) (http://www.cancer.gov). This dataset contained tumor/non-tumor, mutation, copy number variation (CNV), clinical information, and survival information (25 GC patients without survival information) recording as the training set (TCGA-GC) [13]. Subsequently, the GSE167297 (GPL20301 platform) and GSE84426 (GPL6947 platform) were downloaded from Gene Expression Omnibus (GEO) (https://www.ncbi.nlm.nih.gov/geo/). Specifically, the GSE167297 was single cell RNA sequencing (scRNA-seq) dataset comprising 14 gastric tissue samples, of which were 10 GC tissue samples for tumor group and 4 paracancerous tissue samples for normal group [14] (Supplementary Table 1). At the same time, the GSE84426 contained gastric tissue samples from 76 GC patients with transcriptomic data and survival information, which was noted as the validation set [15]. In addition, 279 cell senescence-related genes (CS-RGs) originated from the Database of Cell Senescence Genes (CellAge) (https://genomics.senescence.info/cell/) [16] (Supplementary Table 2).

### The scRNA-seq data analysis

In the GSE167297, The “Seurat” (v 5.0.1) [17] was employed for data quality control, and the rest of the unspecified analyses in this analysis point to this package. Specifically, cells with less than 200 and greater than or equal to 5,000 genes, cells with more than 5% for mitochondrial genes, genes covered by less than 3 cells, and genes with less than or equal to 200 and greater than or equal to 30,000 count numbers were removed. Next, the FindVariable Features function was employed in screening top 2,000 highly variable genes based on variance stabilizing transformation (vst) method, while the LabelPoints function was employed in identifying top 10 highly variable genes. Then, Scale Data function was used to standardize the scRNA-seq data to observe the presence of outliers, and principal component analysis (PCA) was undertaken utilizing Elbowplot function to visualize the results. Meanwhile, the Jackstraw function was utilized to select principal components (PCs) with p < 0.05 in PCA elbow plot for subsequent analyses. Following that, cell cluster analysis was undertaken, whilst results were visualized by Uniform Manifold Approximation and Projection (UMAP) (resolution = 0.4). According to marker genes obtained from published literature [18], annotation analysis of cell clusters was unfolded using DotPlot function to identify cell subpopulation, and the expression of marker genes was observed in distinct cell subpopulations. Afterwards, the proportions of distinct cell subpopulations in the total sample were demonstrated, and cell subpopulations with the highest percentage in the tumor group was defined as the key cells. Besides, to further explore the mechanisms involved in key cells, marker genes from the literature [14, 19] were used to classify and identify subpopulations of key cells, and the expression patterns of marker genes in different cell types were visualized. Moreover, to further understand the process of differentiation of key cells, the “Monocle2” (v 2.30.1) [20] was applied to carry out a simulation analysis of cell trajectory differentiation of key cells. Eventually, to further explore the role of communication between key cells and other cell types, the “CellChat” (v 2.1.2) [21] was applied to perform cell communication analysis on the cell types obtained from the annotation.

### Differential expression analysis and acquisition of candidate genes

To obtain differentially expressed genes (DEGs) in key cells, the FindMarkers function of “Seurat” was applied to screen DEGs between key cells (|log_2_fold change (FC)| ≥ 0.5, p < 0.05), which were denoted as KC-DEGs, and volcano plot of KC-DEGs was drawn by “ggplot2” (v 3.5.1) [22]. Meanwhile, differential expression analysis was carried out by “DESeq2” (v 1.42.0) [23] to acquire GC-DEGs between the tumor and normal groups in TCGA-GC (|log_2_FC| > 0.5, p < 0.05). Subsequently, the results were ordered by the log_2_FC values sorted from largest to smallest, and the “ggplot2” package and “ComplexHeatmap” (v 2.18.0) [24] were employed to draw a volcano map and a heat map. Notably, the top 10 up- and down-regulated GC-DEGs were labelled on the volcano map, and their expression trends were visualized in the heat map. Finally, the “VennDiagram” (v 1.7.3) [25] was applied to gain candidate genes from intersection of KC-DEGs1, GC-DEGs, and CS-RGs.

### Validation of prognostic genes and their potential mechanisms

To identify prognostic genes, based on the TCGA-GC, univariate Cox regression was carried out (p < 0.05) via “survival” (v 3.7.0) (https://CRAN.R-project.org/package=survival), and “forestplot” (v 3.1.3) (https://CRAN.R-project.org/package=forestplot) was utilized to visualize the results. Afterwards, Coxph function of “survival” was used to make proportional hazards (PH) assumption for these genes to gain candidate prognostic genes (p > 0.05). Then, the glmnet package (v 4.1.8) [26] was applied to proceed tenfold cross-validation least absolute shrinkage and selection operator (LASSO) regression analysis. The genes with the optimal model and the regression coefficient not penalized to 0 were selected as prognostic genes.

Subsequently, to estimate the correlation and functional similarity among prognostic genes, cor function of “stats” (v 4.1.3) (https://search.r-project.org/R/refmans/stats/html/00Index.html) was utilized to analyze correlation for prognostic genes via Spearman correlation analysis. Meanwhile, “GOSemSim” (v 2.28.1) [27] was employed to compute functional similarity scores of prognostic genes to explore the synergistic effects of prognostic genes in biological processes. Ultimately, the Wilcoxon test was employed for checking variant expression of prognostic genes among tumor and normal groups (adj. p < 0.05).

### Construction of the risk model

After obtaining prognosis genes, the risk model of prognostic genes was constructed, and each GC patient from the TCGA-GC had their risk score determined via following formula: risk score = $\sum_{\text{i}=1}^{\text{N}}\text{ei}\times \text{wi}$ (N indicated gene count, e indicated expression of prognostic gene, and w indicated coefficient obtained from the LASSO regression analysis). Following that, by optimal cut-off value of risk score, GC patients with survival information in the training set were categorized into high-risk and low-risk groups. Afterwards, survival probability for GC patients of high-risk and low-risk groups was compared utilizing Kaplan–Meier (KM) survival analysis (p < 0.05) by “survminer” and “survival”. Whilst, the receiver operating characteristic (ROC) curves were plotted by “pROC” (v 1.18.5) [28] to assess the predictive value of risk model (area under curve (AUC) > 0.6). Notably, the efficacy of risk model in the GSE84426 was furthermore confirmed based on the same method as above.

To further evaluate the effectiveness of the risk score in prognostic prediction, we compared it with common clinicopathological factors such as age, sex, tumor stage, M-stage, N-stage, and T-stage. We grouped different factors, used the KM method to plot survival curves, and compared the survival differences among different groups through the Log-rank test. The survdiff function was used to conduct the Log-rank test to assess the survival differences among different groups.

Meanwhile, we stratified the data according to the clinical stages (Stage i/ii vs. Stage iii/iv). Then, we separately plotted the KM survival curves for the high- and low-risk groups within each stage, and compared the survival differences among different groups through the Log-rank test.

### Independent prognostic analyses and construction of nomogram

To further explore the potential of risk scores in GC clinical, based on the TCGA-GC, the Wilcoxon test was applied to inspect disparities for risk scores at gender, age, T stage, N stage, stage for GC patients (p < 0.05). Afterwards, for better application, the univariate Cox regression analysis on variables was conducted, namely risk score, gender, age, stage, T stage, N stage and M stage (p < 0.05). Subsequently, the PH assumption test was constructed (p > 0.05), and independent prognostic factors were acquired by retaining clinical indicators in multivariate Cox regression analysis (p < 0.05). Based on this, the “rms” (v 6.5.0) [29] was employed to construct nomogram predicting 3/5/7 years survival rates for GC. Finally, a calibration curve and a ROC curve (AUC > 0.06) were plotted by “Rregplot” (v 1.1) (https://CRAN.R-project.org/package=regplot) and “timeROC” (v 0.4) [30], respectively, to evaluate accuracy for the nomogram model at predicting survival from GC patients.

### Gene set enrichment analysis (GSEA)

Moreover, to further understand the potential functions of the risk groups in GC, based on the risk groups, the “DESeq2” (v 1.42.0) [23] was applied to analyze the differences, whilst log_2_FC of genes was ranked. Afterwards, the background gene set “c2.cp.kegg.v2023.2.Hs.symbols.gmt” was acquired from the Molecular Signatures Database (MSigDB) database (https://www.gsea-msigdb.org/gsea/msigdb), and the clusterProfiler (v 4.10.1) [31] was applied to conduct the GSEA (adj. p < 0.05).

### Analysis of immune infiltration and immunotherapy

To observe changes in the immune microenvironment between risk groups, scores of 28 immune cells [32] among risk groups were computed using ssGSEA algorithm of GSVA (v 1.50.0) [33] and compared utilizing the Wilcoxon test (p < 0.05). Furthermore, the “Corrplot” (v 0.92) [34] was applied to further understand the Spearman correlations between differential immune cells, as well as between differential immune cells and prognostic genes (|cor| > 0.3, p < 0.05).

Besides, the “ESTIMATE” (v 1.0.13) [35] was applied to calculate the immune score, the stromal score and the Estimation of STromal and Immune cells in MAlignant Tumor tissues using Expression data (ESTIMATE) score for each TCGA-GC sample, and the Wilcoxon test was applied to compare the differences in the scores of the risk groups (p < 0.05). Then, based on 68 immune checkpoint genes obtained from the literature [36] (Supplementary Table 3), the Wilcoxon test was employed to compare disparities in expression for immune checkpoint genes between risk groups (p < 0.05), and relationships between prognostic genes and discrepant immune checkpoint genes, as well as between immune checkpoint genes and risk scores, were investigated utilizing Spearman correlation analysis (p < 0.05). Ultimately, to further assess differences in immunotherapy between risk groups, the tumor mutation burden (TMB) and microsatellite instability (MSI) of the samples was analyzed (p < 0.05).

### Mutation analysis and drug sensitivity analysis

Based on the somatic mutation data from the TCGA-GC samples, the Maftools (v 2.18.0) [37] was applied to generate waterfall plots to explore mutation signatures between risk groups. Moreover, to assess differences in drug sensitivity between risk groups, the half-maximal inhibitory concentration (IC_50_) values of 138 drugs in the Genomics of Drug Sensitivity in Cancer (GDSC) (https://www.cancerrxgene.org/) were collected. Followed by the pRRophetic algorithm (v 0.5) [38] was applied to assess the sensitivity of risk groups to chemotherapeutic agents, and the Wilcoxon test was applied to contrast the differences in IC_50_ of drugs between the risk groups (p. adj < 0.05). The top 10 drugs were visualized via “ggplot2”.

### Statistical analysis

Analyses for this study were executed in R software (v 4.2.3), whilst p < 0.05 indicated statistical significance.

## Results

### T cells were key cells, and their communication with other cell types was weakened

**Fig. 1:**
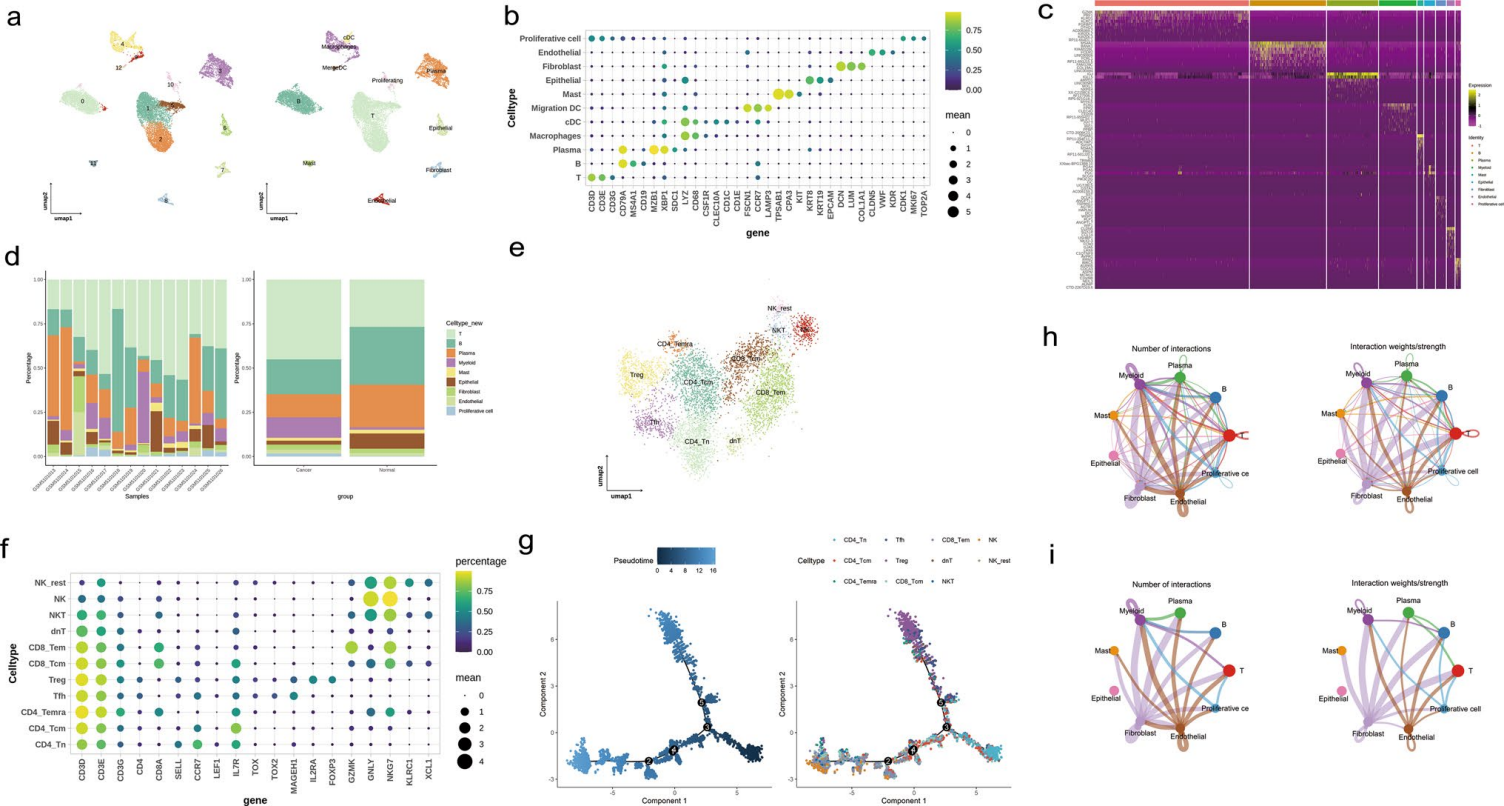
T cells were recognized as key cells, and the trajectories of T cells and intercellular communication were analyzed. **a** UMAP of cell clusters and cell types. The abscissa was the marker genes, and the ordinate was the cell clusters. The size of the bubbles represented the percentage of gene expression, and the color indicated the mean value of gene expression. The gene with the highest gene-expression value in one cluster was found, and it was mapped to the cell type corresponding to the marker. This cell type was the cell type of this cluster. **b** Expression levels of marker genes for each cell type. **c** Heatmap shows the expression of marker gene in all cell types The strips at the top with different colors represented different cell clusters. The ordinate was the gene names. In the main part of the heat-map, the color was used to represent the gene expression levels. The expression levels, from low to high, were divided as follows: purple > black > gold. **d** The proportion of different cells of patients in GC samples. **e** 11 T cell subtypes were classified The points in different colors in the figure represented the different cell clusters obtained through clustering. **f** Expression levels of marker genes for each T cell subtype. **g** Pseudotemporal trajectory analysis revealed the differentiation of T cells into different subpopulations. **h** Number and strength of interactions between cell populations In the figure, different colors represented different cell types. The size of the circles indicated the number of cells, and the thickness of the lines represented the intensity of cell interactions. **i** Number and strength of interactions between the top 25% of cell populations

In the GSE167297, scRNA-seq data had 25,588 cells and 20,730 genes before quality control, while a total of 13,927 cells and 20,730 genes were gained for subsequent analyses after quality control (Supplementary Fig. 1). Then, the top 2,000 highly variable genes, such as IGLL5, TPSAB1 and PGC, were picked to enter the downstream analysis (Supplementary Fig. 2). Afterwards, a gradual stabilization was observed after 20 PCs, indicating that the true signal comes mainly from the first 20 PCs (Supplementary Fig. 3). Next, the cells co-clustering into 13 different cell clusters the marker genes displayed specific high expression in 9 cell types (Table 1), indicating the reliability of the cell annotation results (Fig. 1a, b). Subsequently it was observed that T cells accounted for the highest percentage of GC samples, followed by B cells, thus T cells were used as key cells (Fig. 1c, d). Afterwards, T cells were reorganized into 11 cell clusters annotated into a total of 11 cell types, namely regulatory T cells (Treg), effector memory T cells re-expressing CD45RA (CD4 Temra), T follicular helper cells (Tfh), CD4 Tcm, naive CD4 T cells (CD4 Tn), CD8 Tcm, double negative T cells (dnT), CD8 Tem, natural killer T cells (NKT), NK, rest and NK (Fig. 1e). The specific high expression of marker genes among different T cell types indicated the accuracy of the annotation (Fig. 1f). Based on this, it can be observed that in early T cell development it was predominantly CD4 Tn that differentiates, whereas in later stages it was predominantly Treg and CD8 Tem (Fig. 1g). Surprisingly, fibroblasts and endothelial cells had strong communication with other cell types. Notably, T cells primarily communicated with myeloid cells. However, T cell communication with other cell types was not observed in the top 25% of interactions between cell types, and similarly, neither was mast cells and epithelial cells (Fig. 1h, i). Overall, exploring these interactions will help to understand the biological relevance of cell-to-cell signaling dysregulation in GC, providing a scientific basis for unravelling the molecular mechanisms of the disease and discovering new therapeutic targets.

**Table 1:** 13 cell clusters annotated to 9 cell types

Cell type	Marker gene
T cells	CD3D, CD3E, CD3G
B cells	CD79A, MS4A1, CD19
Plasma	MZB, XBP1, SDC1
Myeloid	LYZ, CD68, CSF1R
Mast	TPSAB1, CPA3, KIT
Epithelial	KRT8, KRT19, EPCAM
Fibroblast	DCN, LUM, COL1A1
Endothelial	CLDN5, VWF, KDR
Proliferative cell	CDK1, MKI67, TOP2A

### The identification of prognostic genes proved to be reliable

**Fig. 2:**
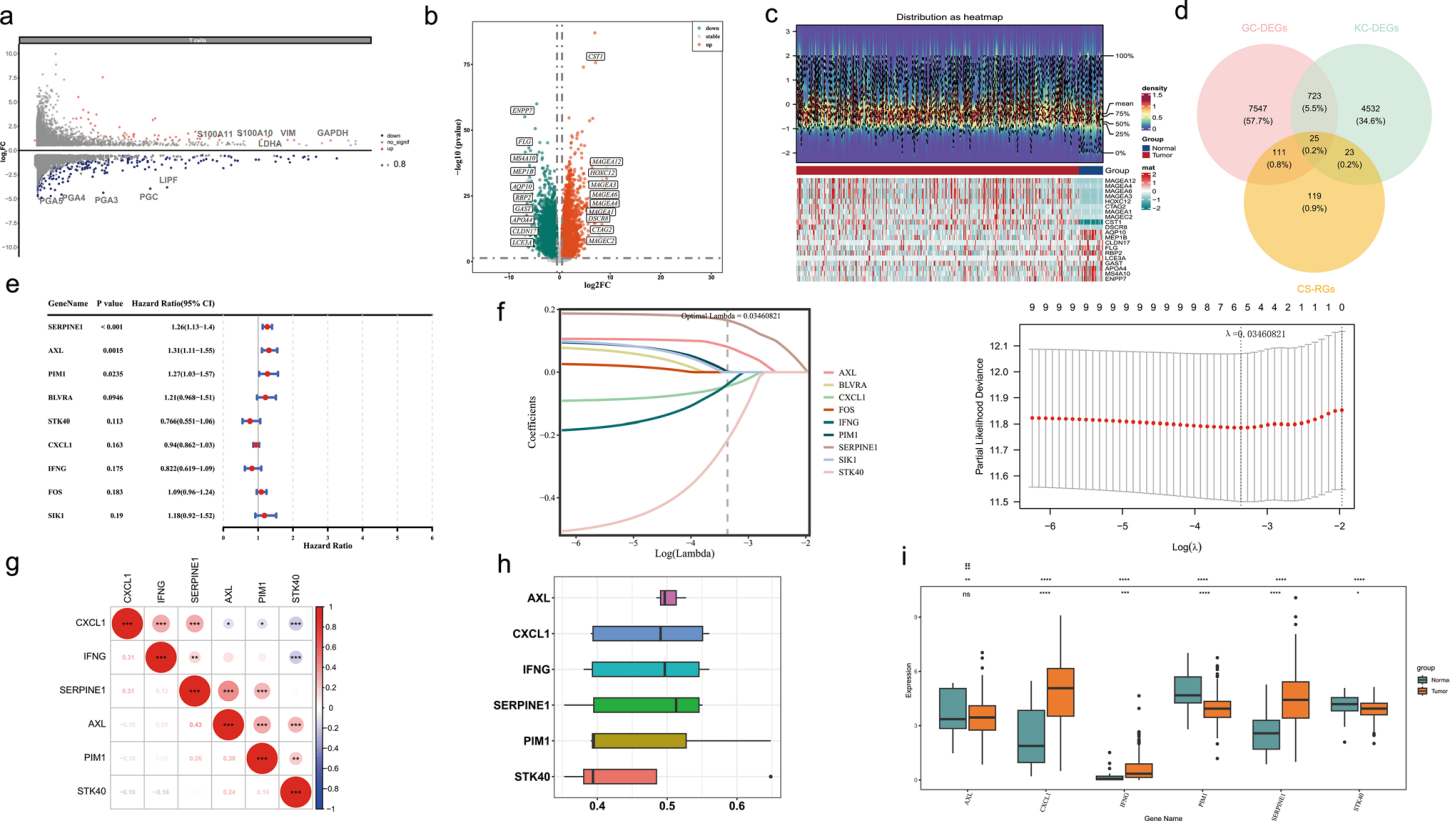
Screening for prognostic genes and the correlation between them, as well as the analysis of their functional correlation. **a** Volcano plot of KC-DEGs among 11 cell types of T cells. **b** Volcano plot of GC-DEGs between GC and control in TCGA. In the figure, the green dots represented the differentially down-regulated genes, and the red dots represented the differentially up-regulated genes. Meanwhile, the top ten up-regulated and down-regulated genes in terms of fold-change were marked. **c** Heatmap of the top 10 up-regulated and down-regulated genes organized by their fold change The picture consisted of two parts. The upper part was a density heatmap of the expression levels of differentially expressed genes in the samples, which showed the lines of five quantiles and the average value. The lower part was a heatmap of the expression of differentially expressed genes in the samples. **d** Intersection of KC-DEGs, GC-DEGs and CS-RGs named TCS-RGs. **e** Univariate cox regression analysis of OS for TCS-RGs, and 9 genes with P < 0.2. **f** LASSO regression of the 9 candidate prognostic genes and Cross-validation for tuning the parameter selection in the LASSO regression. **g** Degree of association between distinct prognostic genes. **h** Degree of similarity in biological functions among the prognostic genes. **i** Box plot visualizing the expression levels of 6 prognostic genes between tumor and normal groups

A total of 5303 KC-DEGs were acquired in T cells, including 1003 up-regulated and 4300 down-regulated KC-DEGs in the tumor group (Fig. 2a). Meanwhile, a total of 8406 GC-DEGs were obtained between tumor and normal groups in the TCGA-GC, which contained 4,155 up-regulated and 4,251 down-regulated GC-DEGs (Fig. 2b, c). Afterwards, 25 candidate genes called TCS-RGs were determined through the intersection of 5,303 KC-DEGs, GC-DEGs, and CS-RGs (Fig. 2d). Based on univariate Cox regression analysis, 9 candidate prognostic genes were obtained by PH assumption (p > 0.05), SERPINE1, AXL, PIM1, BLVRA, STK40, CXCL1, IFNG, FOS, and SIK1 (Fig. 2e) (Table 2). Then, after LASSO regression analysis, 6 prognostic genes were identified (lambda. min = 0.03460821) (Fig. 2f), namely AXL, PIM1, STK40, CXCL1, IFNG, and SERPINE1.

**Table 2:** PH assumptions for 9 candidate prognostic genes

Gene	p
SERPINE1	0.587755187
BLVRA	0.096269757
IFNG	0.651172454
PIM1	0.670735905
SIK1	0.492264844
CXCL1	0.954488602
AXL	0.981585519
FOS	0.785798719
STK40	0.204070286

Interestingly, there were strong positive correlations between prognostic genes and no strong negative correlations. Among them, the most significant positive correlation existed between AXL and SERPINE1 (cor = 0.43, p < 0.001) (Fig. 2g). In addition, prognostic genes had similar functions, such as CXCL1 and IFNG, suggesting that they had some synergistic roles in biological processes (Fig. 2h). Moreover, expression of 5 prognostic genes was remarkably distinct between tumor and normal groups (p < 0.05). Specifically, the CXCL1, IFNG, and SERPINE1 in the tumor group had higher expression, while PIM1 and STK40 in the tumor group had lower expression. The AXL, although also highly expressed in the tumor group, did not reach significance levels (Fig. 2i).

### Patients with GC at high risk had lower survival rates

**Fig. 3:**
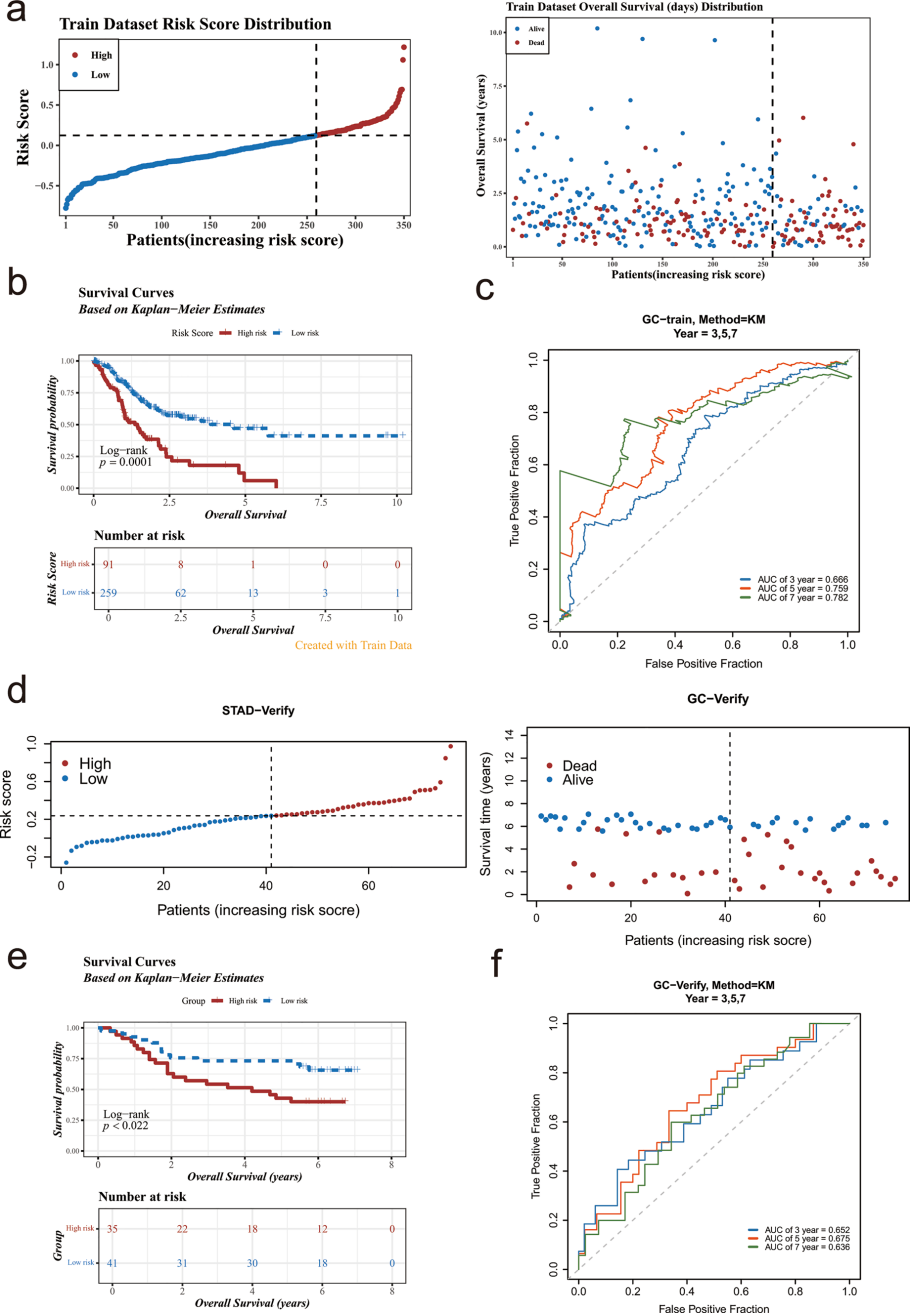
The construction and validation of the risk model in the TCGA and GEO cohorts. **a** Distribution and survival status of patients based on the risk score On the left-hand side figure, the abscissa was the patient samples sorted according to the risk scores from low to high, with the risk scores increasing successively from left to right. The ordinate was the risk score. The dotted line represented the optimal value of the risk score and the corresponding number of patients; On the right-hand side figure, the abscissa was also the patient samples sorted according to the risk scores, with the risk scores increasing successively from left to right. The ordinate was the survival time. The dotted line represented the optimal value of the risk score and the corresponding number of patients. **b** Kaplan–Meier curves for the OS of patients in the high- and low-risk groups Red represented the high-risk group, and blue represented the low-risk group. The abscissa was time, and the ordinate was the survival rate. **c** ROC curves demonstrated the predictive efficiency of the risk score The area under the curve was called AUC (Area Under Curve), which was used to represent the prediction accuracy and sensitivity. The higher the AUC value, that is, the larger the area under the curve, the higher the prediction accuracy. **d** Distribution and survival status of patients in the GEO cohort. **e** Kaplan–Meier curves for comparison of the OS between low- and high-risk groups (GSE84426). **f** Time-dependent ROC curves for GCs (GSE84426)

The risk model was constructed through 6 prognostic genes and their coefficients (Table 3). According to optimal cutoff value (0.2369964) GC samples were classified into high-risk (n = 91) and low-risk groups (n = 259). The results displayed that the mortality count escalated with the rising risk scores (Fig. 3a). Thereafter, a marked difference in survival between risk groups in the KM curves (p = 0.0001), patients classified in the high-risk group exhibited a lower probability of survival (Fig. 3b). Furthermore, the AUCs of 3, 5 and 7 years were 0.666, 0.759 and 0.782, suggesting that the risk model constructed based on prognostic genes had great ability to predict GC survival (AUC > 0.6) (Fig. 3c). Ultimately, it should be noted that in the GSE84426, according to the optimal threshold (0.2369964), the GC samples were categorized into high-risk (n = 35) and low-risk groups (n = 41). The rest of the consequences were accord with the TCGA-GC, suggesting the reliability of the risk score model constructed based on AXL, PIM1, STK40, CXCL1, IFNG, and SERPINE1 (Fig. 3d–f).

**Table 3:** Coefficients for prognostic genes

Gene	Coefficient
AXL	0.081104755
PIM1	0.001269498
STK40	-0.215613352
CXCL1	-0.044675072
IFNG	-0.038825602
SERPINE1	0.16550985

To further evaluate the effectiveness of the risk score in prognostic prediction, we compared it with common clinicopathological factors such as age, sex, tumor stage, M-stage, N-stage, and T-stage. We used the rank-sum analysis method to analyze the relationship between each factor and prognosis. The results showed that the p-values of the Log-rank tests for age, M-stage, and N-stage were all less than 0.05, indicating significant survival differences within the age, M-stage, and N-stage groups. However, none of these differences were as significant as those in the risk-score group (Supplementary Fig. 4). A stratified analysis was carried out according to tumor stages (Stage i/ii and Stage iii/iv). It was found that both stratifications (Stage i/ii and Stage iii/iv) demonstrated significant differences in survival prediction by the risk score. Moreover, the p value in the Stage i/ii group (p = 0.0001) was lower than that in the Stage iii/iv group (p = 0.0023), indicating that the prognostic effect of the risk score might be more significant within the Stage i/ii group (Supplementary Fig. 5).

### Nomogram constructed based on risk scores and age was favorable predictors of survival in GC

**Fig. 4:**
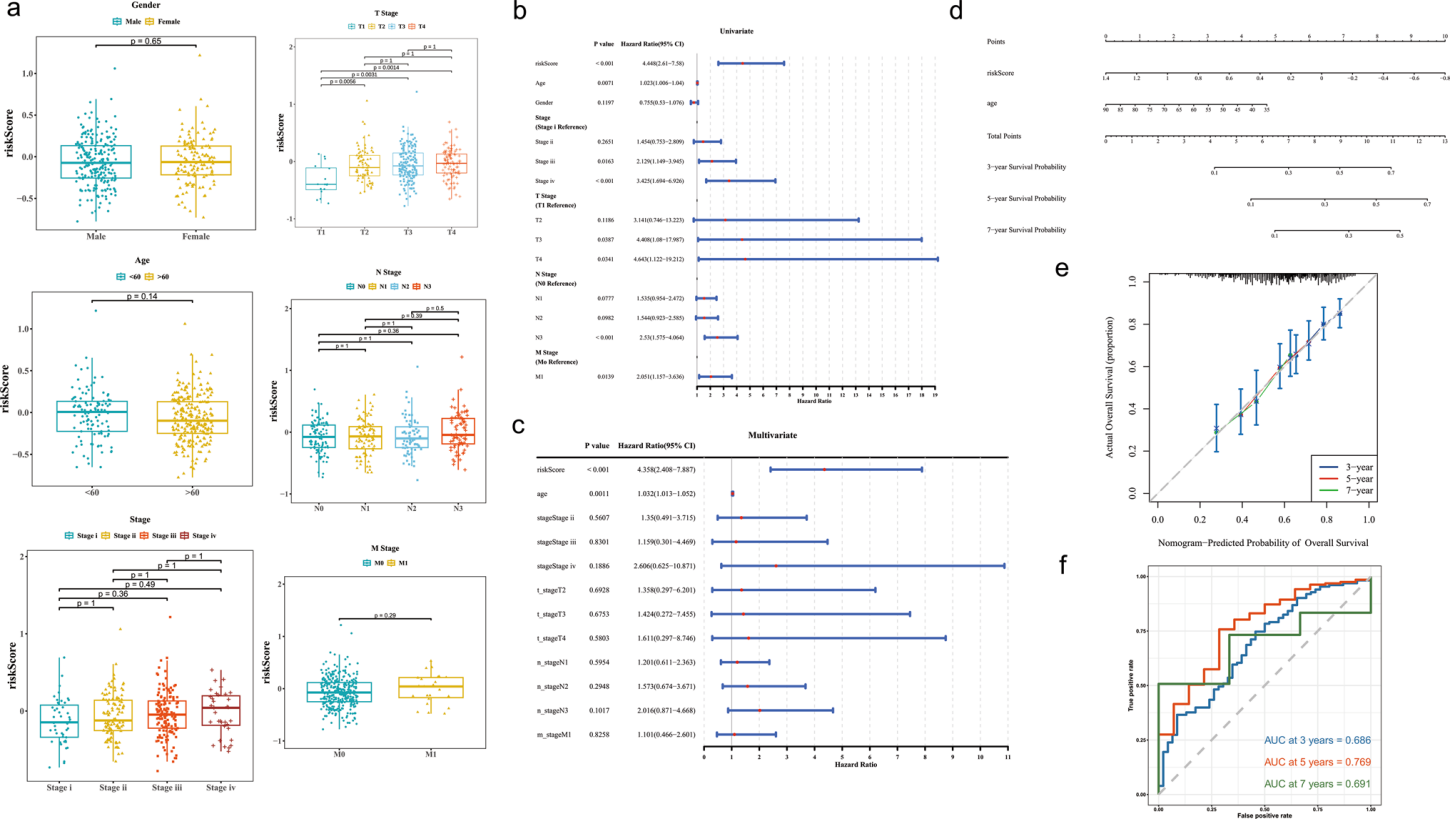
Correlation analysis of risk score with clinical features and nomogram establishment and GSEA analysis between risk groups. **a** Relationship between gender, age, tumor stage, T stage, N stage, M stage with the analysis model. **b** Univariate Cox analysis of risk scores and clinical characteristics. **c** Multifactorial Cox analysis. **d** Construction of the nomogram model. **e** The calibration curve of the nomogram The points on the calibration curve represented the predicted survival probabilities by the model and the actually observed survival probabilities. The vertical lines corresponding to the points on the calibration curve represented the confidence intervals at those positions. The blue crosses on the calibration curve represented the results after the stratified Kaplan–Meier correction for each point. **f** The AUC of the prediction of 3, 5, and 7-year survival rates of GC

Based on the TCGA dataset, combined with the clinical characteristics of the tumors, we observed significant differences in risk scores in T1 and T2 (p < 0.0058), T1 and T3 (p < 0.0031) and T1 and T4 (p < 0.0014). Notably, the risk scores increased with the progression of T stage, indicating the association between risk scores and GC progression. While there were no significant differences in clinical characteristics such as age and gender (p > 0.05) (Fig. 4a). Subsequently, a total of 2 independent prognostic factors were obtained, namely risk scores and age (Table 4) (Fig. 4b, c). Based on this, a nomogram was constructed to predict GC survival, which showed an increased in 3, 5, and 7-year survival in GC patients as the Total Points increased (Fig. 4d). Besides, the calibration curve displayed that the slope was close to 1, and the AUCs were 0.686, 0.769 and 0.691 of 3, 5, and 7 years, demonstrating the favorable accuracy of the nomogram (AUC > 0.6) (Fig. 4e, f). Overall, the nomogram constructed based on independent prognostic factors had favorable prediction in GC survival, which supplied an effective individualized decision-making tool for the clinical aspects of the GC.

**Table 4:** PH assumption for candidate independent prognostic variables

Id	P value
riskScore	0.743613809561138
age	0.606761269821429
stage	0.379914012059854
t_stage	0.554134833759905
n_stage	0.524518387848716

### Different critical pathways were involved between risk groups

**Fig. 5:**
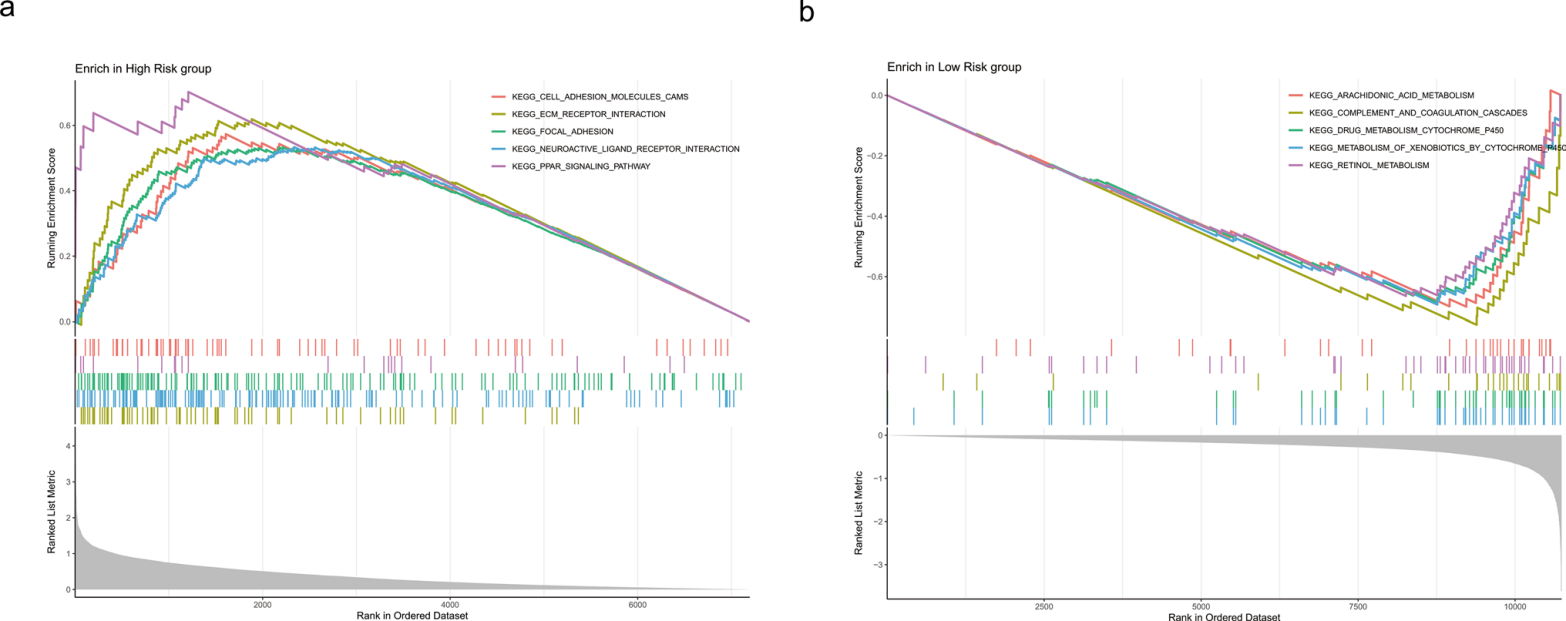
GSEA analysis between risk groups. **a** GSEA analysis of KEGG in high risk group. **b** GSEA analysis of KEGG in low risk group The figure was divided into three parts. The upper part showed the calculation process of the Enrichment Score (ES) values. Starting from the left and moving to the right, an ES value was calculated for each gene, and these values were connected to form a line. There was a particularly prominent peak on the far left/right side, which was the ES value of the gene set phenotype. The middle part had each line representing a gene in the gene set and its ranking position in the gene list. The lower part was the distribution map of the rank values of all genes. The vertical axis was the ranked list metric, which was the value of the gene's ranking quantity. It could be understood as the fold-change value after "formula-based" processing

Based on the TCGA dataset, we further explored the possible pathways involved in risk groups through GSEA. Among them, the high-risk group was significantly enriched for a total of 26 entries, such as ECM receptor interaction, neuroactive ligand receptor interaction and PPAR signaling pathway (Fig. 5a), while the low-risk group was significantly enriched for a total of 23 pathways, such as drug metabolism-cytochrome P450, complement and coagulation cascades and retinol metabolism (Fig. 5b) (Supplementary Table 4). Through GSEA, pathways involved in risk groups were revealed, providing insights into personalised medicine for GC clinical stratification.

### Higher risk group might be associated with lower immunotherapy benefit

**Fig. 6:**
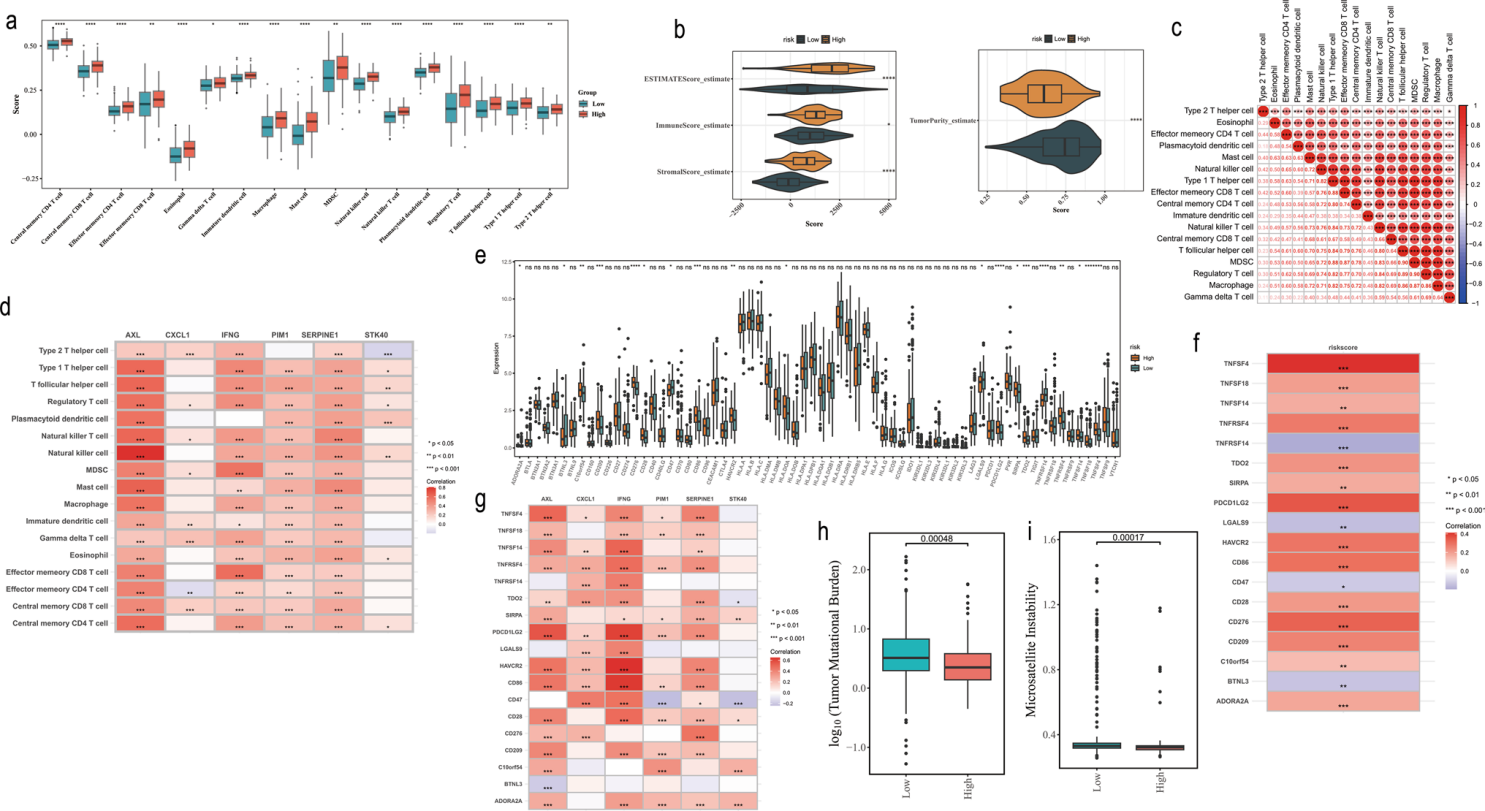
Immunoinfiltration and immunotherapy analyses were conducted between risk groups. **a** Box plot depicting the immunoinfiltration of 17 immune cells between high and low-risk groups. **b** Box plot of immune microenvironment and cell purity scores of cells between high and low-risk groups. **c** Correlation analysis of risk scores with significantly different immune cells. **d** The relationship between differential immune cell populations and prognostic genes. **e** Box plot visualizing the expression levels of immune checkpoint genes between high-risk and low-risk groups. **f** The relationship between immune checkpoint genes and risk scores In the figure, the color red represented a positive correlation, blue represented a negative correlation, and white represented no correlation. **g** The relationship between immune checkpoint genes and prognostic genes In the figure, the color red represented a positive correlation, blue represented a negative correlation, and white represented no correlation. **h** Differences in TMB (Tumor Mutation Burden) between the high-risk and low-risk groups. **i** Differences in MSI (Microsatellite Instability) between the high-risk and low-risk groups

Based on the TCGA dataset, there were 17 differential immune cells in the risk groups, surprisingly, all of them had higher scores in the high-risk group (Fig. 6a). This implied that the tumor microenvironment in the high-risk group of gastric cancer had a higher level of infiltration of stromal and immune cells, and it was further verified in the ESTIMATE analysis. Specifically, the ESTIMATE score, immune score and stroma score were significantly higher in the high-risk group than in the low-risk group, which also corresponded to the high-risk group having low tumor purity (Fig. 6b). Subsequently, correlations between differential immune cells and their association with prognostic genes were revealed. Notably, significant positive correlations were found among 17 differential immune cells, and the strongest positive correlations were found between MDSC with T follicular helper cell (cor = 0.90, p < 0.001) and regulatory T cell (Tregs) (cor = 0.90, p < 0.001) (Fig. 6c). Afterwards, AXL, IFNG, PIM1, and SERPINE1 had remarkable positive correlations with basically 17 differential immune cells, and the greatest positive association among natural killer cells and AXL was discovered (cor = 0.81, p < 0.001) (Fig. 6d) (Supplementary Table 5).

Moreover, in the immunotherapy analysis, the expressions of 19 immune checkpoint genes were noticeably disparities among the groups at high and low risk, and remaining differential immune checkpoint genes had higher expression in the high-risk group except for BTNL3, CD47, LGALS9, and TNFRSF14 (Fig. 6e). Notably, HLA.DOA had no expression data in the training set, thus the remaining 18 differential immune checkpoint genes were used for subsequent analyses. Meanwhile, except for TNFRSF14, LGALS9, CD47, and BTNL3 (negative correlations with risk score), the remaining differential immune checkpoint genes had positive association with risk score (Fig. 6f) (Supplementary Table 6). Similarly, a largely positive correlation between prognostic genes and differential immune checkpoint genes was also observed, with the IFNG had the largest positive association with HAVCR2 (cor = 0.65, p < 0.001) (Fig. 6g) (Supplementary Table 7). Ultimately, the results of the TMB and MSI analysis showed that the TMB and MSI were lower in the high-risk group compared to the low-risk group (Fig. 6h, i), which might imply that patients in the high-risk group were less immunogenic and might have a weaker response to immunotherapy and thus had a lower treatment benefit.

### High-risk groups had higher mutation rates and access to drugs with low IC50

**Fig. 7:**
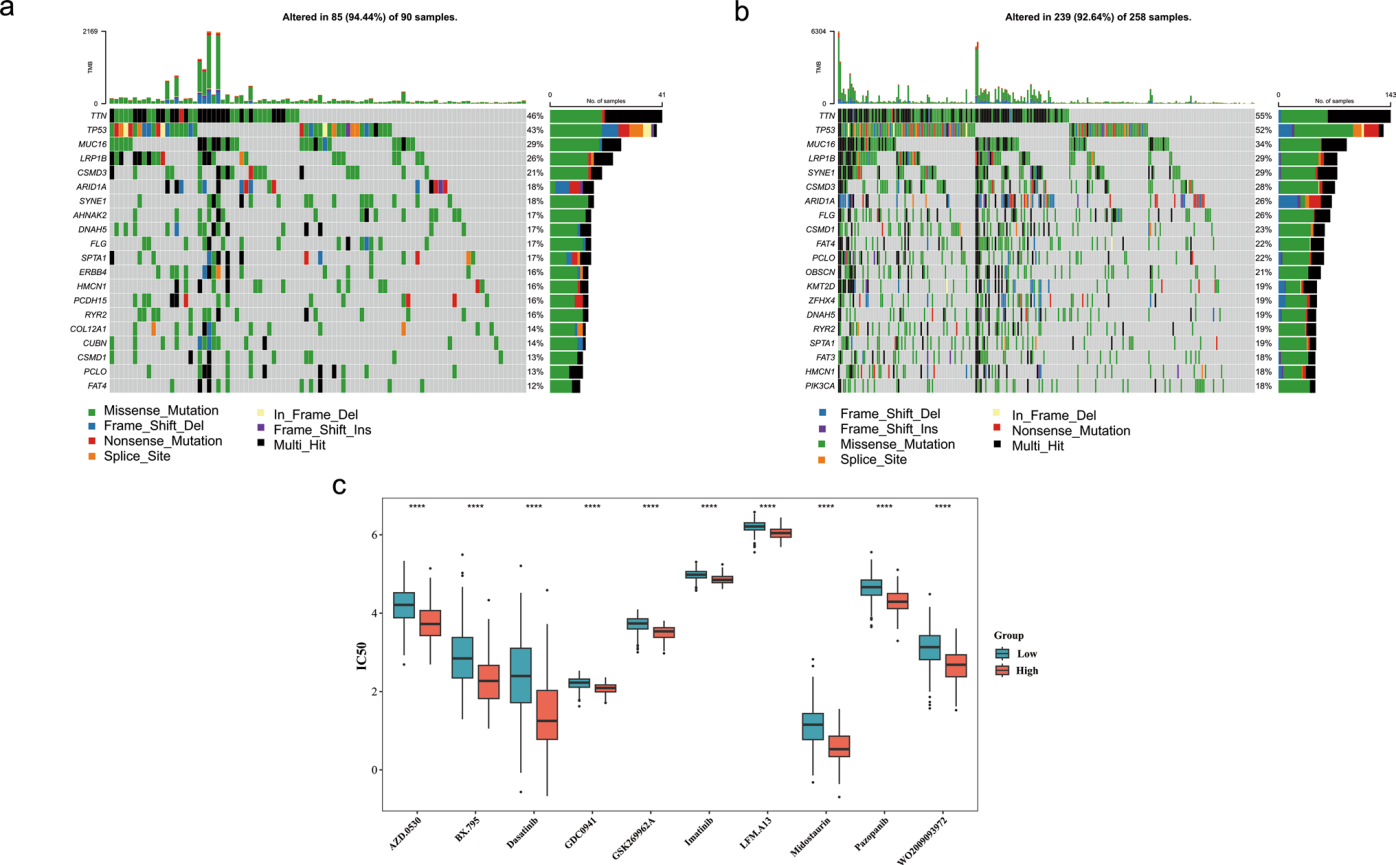
Comparative analysis of drug sensitivity and somatic mutations was conducted between risk groups. **a** Waterfall map of somatic mutations in high-risk groups This figure was divided into four parts. The colors and annotations in the lower-left corner represented the mutation types. The histogram in the upper part represented the Tumor Mutational Burden (TMB), which was used to evaluate the number of somatic mutations occurring in the coding region of the tumor cell genome. **b** Waterfall map of somatic mutations in low-risk groups. **c** Box plot visualizing the drug sensitivity analysis between high and low-risk groups

The probability of mutation was higher in the high-risk group (94.44%) than that of in the low-risk group (92.64%). Both groups had predominantly missense mutation and multiple hits (multi hit), and the genes predominantly mutated in the risk groups were TTN and TP53 (Fig. 7a, b). The high mutation rate reflected the instability of the tumor genome and suggested an association between high-risk groups and GC progression. Moreover, to assess variation in drug sensitivity among high-risk and low-risk groups, 138 drug sensitivities were inspected. Subsequently, there were 90 drugs that were markedly different among high-risk and low-risk groups (Supplementary Table 8), and top 10 differential drugs had lower IC_50_ values in the high-risk group (Fig. 7c), such as GSK269962A, pazopanib, midostaurin. This demonstrated the potential of these drugs in the clinical stratification of GC patients.

## Discussion

GC ranks among the most crucial malignancies worldwide, accompanied by a poor prognosis and posing a grave threat to human health [39]. Previous study demonstrated that TCS was capable of inducing a weakening of the immune system's defenses against diseases such as infections and cancer [40]. However, few studies have elucidated the mechanism of TCS-related genes in GC. Consequently, based on the public database, this study identified six prognostic genes (AXL, PIM1, STK40, CXCL1, IFNG, and SERPINE1) relevant to TCS in GC through a sequence of bioinformatics analyses and established prognostic risk models founded upon these genes, presenting a fresh perspective for facilitating the progress of GC targeted therapy and elevating the survival rate of patients.

Several studies have shown that AXL is a member of the TAM family of receptor tyrosine kinases (RTKs). Cancer-associated fibroblasts (CAFs) can enhance the aggressiveness of GC cells by activating the GAS6/AXL signaling axis [41]. PIM1 is a serine/threonine kinase. Its high expression in gastric adenocarcinoma is related to lymph node metastasis, suggesting its potential as a diagnostic biomarker, prognostic indicator, and therapeutic target [42, 43]. STK40 (serine/threonine kinase 40), also known as SHIK, negatively regulates NF-κB transcription [44]. Studies show that STK40 may be linked to unfavorable prognosis in gastric and bladder cancers due to its role as a cellular senescence gene [45, 46]. CXCL1, a member of the chemokine family, is related to tumor cell invasion (stage T2-T4), lymph node metastasis, venous invasion, peritoneal cytological manifestations, peritoneal metastasis, and CXCR2 expression in stromal cells in the GC microenvironment, showing its role in tumor progression and prognosis [47] [48]. INFG, the interferon gamma gene, encodes a soluble cytokine of type II interferon, a crucial factor influencing immunotherapy responsiveness. IFN-γ upregulates programmed death ligand-1 on tumor cells and reduces cytotoxicity in GC [49]. SERPINE1 is an oncogene that participates in the proliferation and metastasis of GC cells, angiogenesis, immune infiltration, and the tumor inflammatory microenvironment, and is correlated with the poor prognosis of GC [50, 51]. These results confirm the reliability of selected genes and are consistent with the predictive model.

Our research discovered that the survival rate of the high-risk group was lower than that of the low-risk group by establishing a risk model. The ROC curve indicates that the model may possess a favorable prediction effect. The study of the correlation between the model and clinicopathological features revealed an association between risk score and stage T classification. We also identified independent impacts of age and risk score on overall survival in GC patients. These results contributed to the establishment of a nomogram model, and its prediction accuracy was verified by calibration curves. Previous studies have shown that age is a significant predictor for the survival of GC patients, and the prognosis of elderly patients with GC is poorer than that of non-elderly patients [52]. Multivariate analysis showed that both age and wall penetration (T) were independent prognostic factors [53]. Our study aligns with previous findings, therefore, early tumor detection, diagnosis, and treatment are crucial for improving patient survival.

GSEA results indicated that the high-risk group was significantly enriched in the ECM Receptor Interaction pathways. In cancer, the abnormal activation or dysregulation of the ECM Receptor Interaction pathway is intimately associated with the occurrence, development, and metastasis of tumors [54]. For instance, investigations have revealed that the gene expression patterns in the ECM Receptor Interaction pathway are strongly correlated with the prognosis of colorectal cancer [55]. Furthermore, in lung adenocarcinoma, the expression levels of certain genes in this pathway were significantly correlated with patient survival [56]. It is postulated that the ECM Receptor Interaction pathway might play an equally vital role in GC.

We evaluated immune cell infiltration, immune checkpoint expression, TMB, and MSI from various perspectives. Our conclusion suggests that patients with elevated risk scores might not benefit from immunotherapy and that risk scores might serve as prospective predictors for GC patients undergoing immunotherapy. In this study, we found that MDSC had a significant positive correlation with T follicular helper cells and Tregs. Consistent with previous findings that MDSC promotes tumor growth by suppressing the anti-tumor immune response and/or inducing immunosuppressive cells [57], MDSC could be a potential therapeutic target. Meanwhile, we also found that Natural killer cell (NK cell) was one of the differentiating immune cells between high and low risk groups. NK cells are a type of crucial immune cells, belonging to innate lymphocytes. They exert a vital role in the immune defense of the body, and their functional deficiencies are the essential conditions for tumor immune escape [58]. Previous studies have shown that the density of NK cells in GC tissues is an independent prognostic factor affecting the overall survival and disease-free survival of GC patients and can predict the survival rate [59]. Guo et al. discovered that in GC, the killing capacity of NK cells can be augmented by the overexpression of death associated protein kinase 1 (DAPK1), thereby mitigating the immune escape of cancer cells and diminishing the apoptosis of NK cells [60]. Our study found that NK cells had the most significant positive correlation with the prognostic gene AXL, so further studies can be conducted on how the AXL gene affects the process of GC through NK cell.

Numerous studies have demonstrated that significant immune cell infiltration is often associated with favorable clinical outcomes [61]. However, our findings indicate that the high-risk group exhibits a poorer prognosis. This may be attributed to the elevated abundance of immunosuppressive cells, such as Tregs and MDSCs within this group. These cells create an immunosuppressive microenvironment, undermining anti-tumor immunity despite high immune cell infiltration [62]. Moreover, prognostic genes associated with the high-risk group may also impair immune cell function. Additionally, upregulated immune checkpoint genes in this group significantly inhibit immune cell activity. Although there is substantial immune cell infiltration into the tumor microenvironment, the overactivation of immune checkpoints, particularly the enhanced PD-1/PD-L1 signaling pathway, suppresses the function of effector T cells, preventing them from effectively mounting an anti-tumor response [63]. This imbalance in immunomodulatory mechanisms enables tumors to evade immune surveillance, even in the presence of abundant immune cells [64]. In summary, while the high-risk group shows higher immune cell infiltration, factors like immune cell characteristics, prognostic gene functions, and immune regulatory mechanisms result in poor prognosis.

Drug analysis identified 90 drugs with significant differences between high and low risk groups, such as Pazopanib, Midostaurin, etc. Pazopanib is a multi-target tyrosine kinase inhibitor which plays a significant role in the treatment of multiple cancers [65]. For instance, in GC, it can combine with chemotherapy agents to enhance the chemotherapy effect [66]. In renal cell carcinoma (RCC), Pazopanib inhibited the growth of RCC cell line ACHN by promoting cell senescence [67]. Midostaurin, is also such an inhibitor, which is mainly utilized in the treatment of acute myeloid leukemia (AML), particularly in patients with FLT3 mutations [68, 69]. However, it has also been reported in other cancer treatments, for instance, it can enhance the immunotherapy effect of anti-programmed death protein 1 (PD-1) in colon cancer through modulating the TME [70]. However, there are few studies on these drugs in GC. Further research on the efficacy of these drugs in GC may provide new ideas for the treatment of GC.

## Conclusion and limitation

In this study, six prognostic genes (AXL, PIM1, STK40, CXCL1, IFNG, SERPINE1) related to TCS in GC were identified. A risk model and a nomographic model were constructed to predict the survival of GC patients. Additionally, the risk score may be a potential independent prognostic factor, closely associated with the immune microenvironment and clinicopathological features. The results suggest potential evidence for the stratification and precise treatment of GC patients by accurately differentiating survival outcomes and immunotherapy responses. However, this study has the following limitations: 1. The sample size did not encompass all relevant groups, potentially compromising the representativeness of the results. Any bias in sample selection may affect the generalizability of our findings. 2. There was a lack of comprehensive experimental validation, including insufficient testing in cell lines and tissue samples, as well as limited functional experiments. 3. Further research is required to elucidate the interactions between prognostic genes, differentially expressed immune cells, and immune checkpoint genes. In the future, we plan to build a comprehensive single-cell gastric cancer database through global collaborations. This will cover various subtypes and individual differences, providing robust data for identifying key cells and genes linked to immune aging. We will also study molecular mechanisms and functional genomics using CRISPR-Cas9 to screen and validate critical prognostic genes. Knockout and overexpression experiments in cell lines and animal models will help us observe gene function changes on cellular behavior (e.g., proliferation, apoptosis, migration) and the tumor immune microenvironment, identifying regulatory mechanisms across risk groups. Additionally, we will conduct prospective clinical trials to explore personalized treatments based on risk models and genetic profiles, evaluating their safety and efficacy to support clinical translation.

## Supplementary Information

Supplementary Fig. 1 The quality control of scRNA-seq **(a)** Before quality control **(b)** After quality control (TIF 24977 KB)

Supplementary Fig. 2 The variance diagram shows the variation of gene expression in GSE167297 (TIF 13757 KB)

Supplementary Fig. 3 Highly variable genes were analyzed through PCA **(a)** PCA showed that there were no outlier samples in GSE167297 **(b**, **c)** PCA identified the top 20 PCs at P < 0.05 (TIF 36492 KB)

Supplementary Fig. 4 Kaplan–Meier survival curves illustrating the association between clinicopathological factors and risk score in patients **(a)** Kaplan–Meier survival curves of patients with different age groups and their association with risk score **(b)** Kaplan–Meier survival curves of male and female patients and their association with risk score **(c)** Kaplan–Meier survival curves of patients with different clinical stages and their association with risk score **(d)** Kaplan–Meier survival curves of patients with different M-stages and their association with risk score **(e)** Kaplan–Meier survival curves of patients with different N-stages and their association with risk score **(f)** Kaplan–Meier survival curves of patients with different T-stages and their association with risk score (TIF 17369 KB)

Supplementary Fig. 5 Stratified analysis of the prognostic significance of risk scores based on tumor stages (**a**) Survival curves of high- and low-risk groups in stage i/ii based on Kaplan–Meier estimates (**b**) Survival curves of high- and low-risk groups in stage iii/iv based on Kaplan–Meier estimates The red line represented the overall survival probability curve for the high-risk group; the blue dashed line represented the overall survival probability curve for the low-risk group (TIF 6853 KB)

Supplementary Table 1 Sample grouping information within the single cell dataset GSE167297 (XLSX 10 KB)

Supplementary Table 2 279 cell senescence-related genes (CS-RGs) originated from the Database of Cell Senescence Genes (CellAge) (XLSX 15 KB)

Supplementary Table 3 68 immune checkpoint genes obtained from the literature (XLSX 11 KB)

Supplementary Table 4 All the pathways in the high risk and low risk groups (XLSX 16 KB)

Supplementary Table 5 Correlation between prognostic genes and differential immune cells (XLSX 15 KB)

Supplementary Table 6 Correlation between differential immune checkpoint genes and risk scores (XLSX 11 KB)

Supplementary Table 7 Correlation between prognostic genes and differential immune checkpoint genes (XLSX 15 KB)

Supplementary Table 8 Drug sensitivity among high-risk and low-risk groups (XLSX 16 KB)

## Data Availability

All datasets analyzed for this study can be found in the Gene Expression Omnibus (GEO) database (https://www.ncbi.nlm.nih.gov/geo/), the Cancer Genome Atlas (TCGA) (http://www.cancer.gov) and the Database of Cell Senescence Genes (CellAge) (https://genomics.senescence.info/cell/). Relevant data for the secondary analysis were available in the manuscript or in the supplementary information document.
