# Supplementary figures and images for: Identification and validation of T cell senescence-related prognostic genes in gastric carcinoma and investigation of their potential regulatory mechanisms
Source: Discov Oncol. 2025 Jun 2;16:986. doi: 10.1007/s12672-025-02477-4 (PMC12130386; doi:10.1007/s12672-025-02477-4)

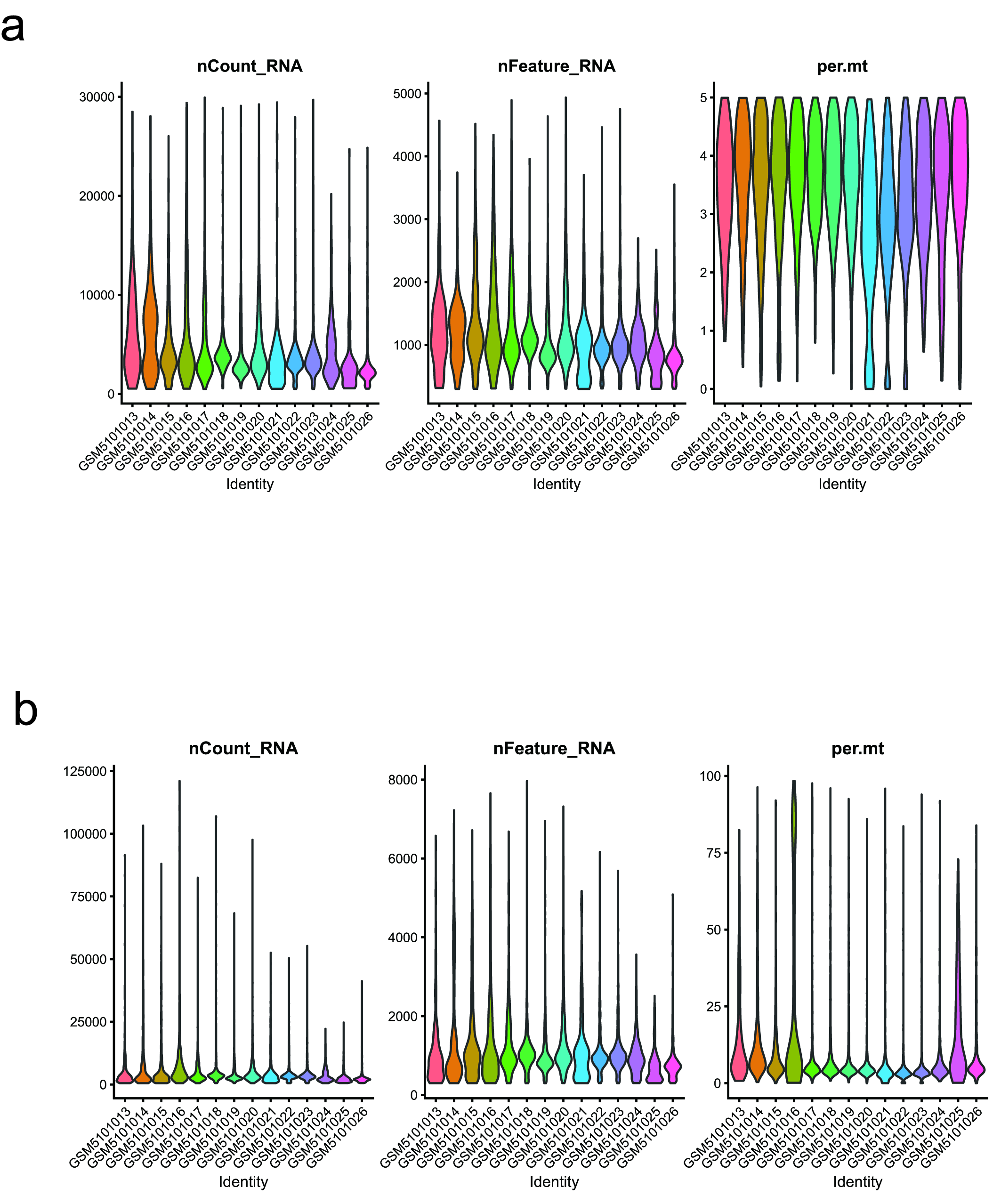

Supplement: Supplementary file 1 — The quality control of scRNA-seq (a) Before quality control (b) After quality control (TIF 24977 KB) [file 12672_2025_2477_MOESM1_ESM.tif]

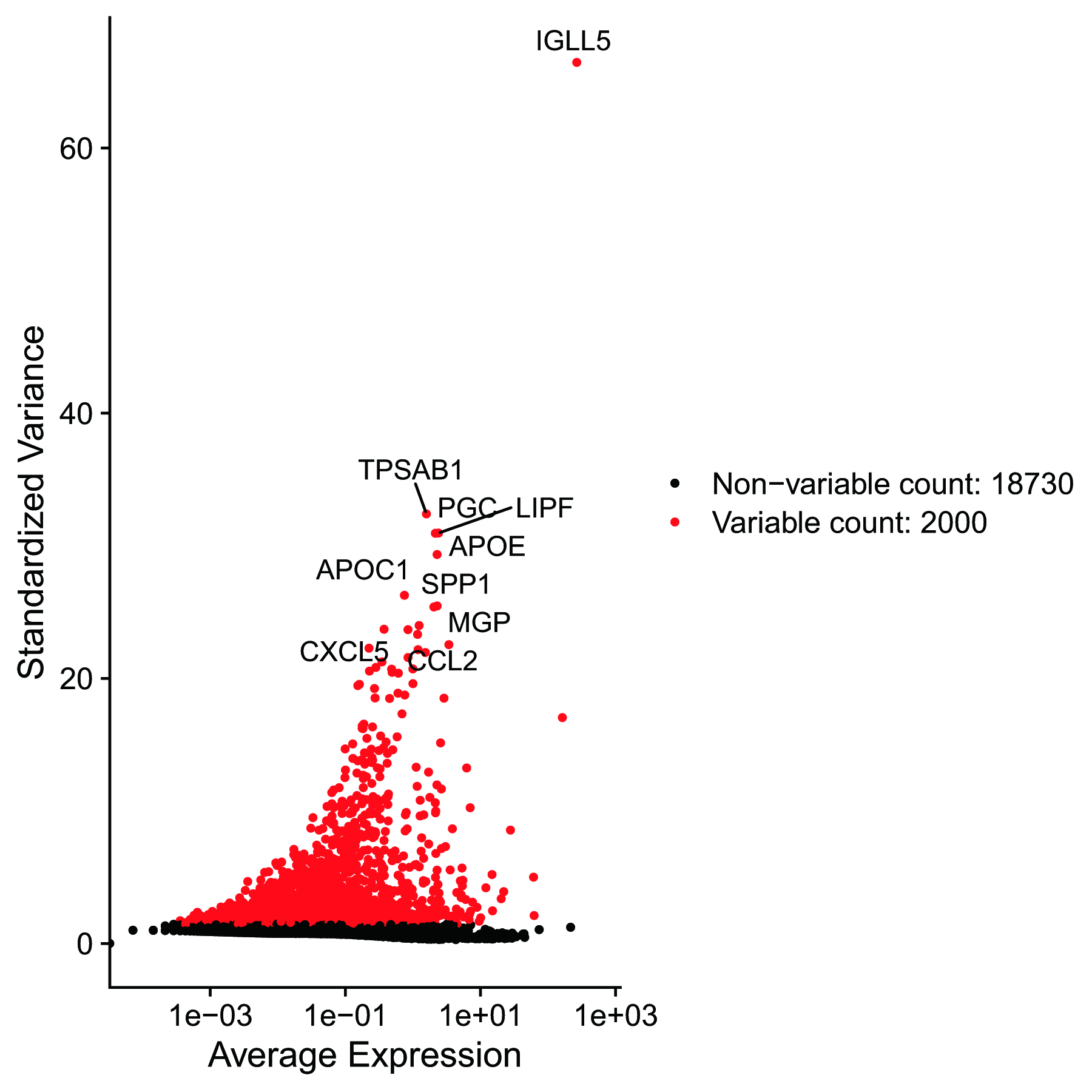

Supplement: Supplementary file 2 — The variance diagram shows the variation of gene expression in GSE167297 (TIF 13757 KB) [file 12672_2025_2477_MOESM2_ESM.tif]

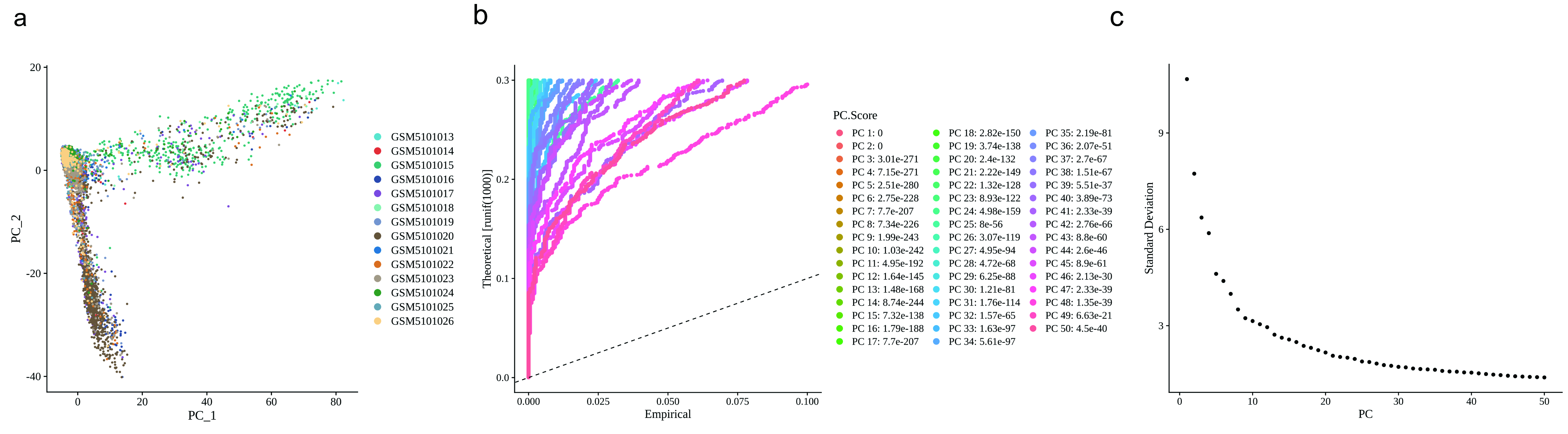

Supplement: Supplementary file 3 — Highly variable genes were analyzed through PCA (a) PCA showed that there were no outlier samples in GSE167297 (b, c) PCA identified the top 20 PCs at P < 0.05 (TIF 36492 KB) [file 12672_2025_2477_MOESM3_ESM.tif]
